# Which is the best predictor of clinically relevant pancreatic fistula after pancreatectomy: drain fluid concentration or total amount of amylase?

**DOI:** 10.1002/ags3.12471

**Published:** 2021-05-11

**Authors:** Yasuyuki Fukami, Takuya Saito, Takaaki Osawa, Takaaki Hanazawa, Takehiro Kurahashi, Shintaro Kurahashi, Tatsuki Matsumura, Shunichiro Komatsu, Kenitiro Kaneko, Tsuyoshi Sano

**Affiliations:** ^1^ Division of Gastroenterological Surgery Department of Surgery Aichi Medical University Nagakute Japan

**Keywords:** distal pancreatectomy, drain amylase, pancreatectomy, pancreatic fistula, pancreaticoduodenectomy

## Abstract

**Aim:**

Drain fluid amylase concentration (DFAC) has been reported as a predictor of clinically relevant postoperative pancreatic fistula (CR‐POPF) after pancreatectomy. However, the clinical significance of measuring the total drain fluid amylase amount (DFAA) considering the daily drainage volume of CR‐POPF remains unclear.

**Methods:**

Data from 216 consecutive patients who underwent pancreaticoduodenectomy (PD) (n = 126) or distal pancreatectomy (DP) (n = 90) between August 2014 and November 2020 were reviewed. All drains were closed but not suctioned. DFAA was calculated by multiplying the DFAC and daily drainage fluid volume. DFAC and DFAA were recorded on d 1 and 3 after pancreatectomy. The cutoff value of CR‐POPF was determined using the receiver operating characteristic curve.

**Results:**

CR‐POPF was found in 75 patients (35%) (PD: 30%, DP: 41%, *P* = .111); the mortality rate was zero. The cutoff value of DFAC‐day 1 was 1757 U/L (sensitivity [SE]: 84%, specificity [SP]: 62%, and accuracy [AC]: 69%). The cutoff value of DFAA‐day 1 was 139 U (SE: 71%, SP: 72%, and AC: 71%). The cutoff value of DFAC‐day 3 was 1044 U/L (SE: 73%, SP: 79%, and AC: 78%). The cutoff value of DFAA‐day 3 was 21 U (SE: 68%, SP: 72%, and AC: 70%). Multivariate analysis indicated that a nondilated pancreatic duct and high DFAC‐day 3 were independently associated with CR‐POPF after PD, indicating that a prolonged operative duration, massive blood loss, and high DFAC‐day 3 are independently associated with CR‐POPF after DP.

**Conclusion:**

DFAC is more reliable than DFAA for predicting CR‐POPF after both PD and DP.

## Graphical abstract

This study aimed to investigate the drain fluid amylase concentration (DFAC) and the drain fluid amylase amount (DFAA) as strong predictors of clinically relevant postoperative pancreatic fistula (CR‐POPF) following pancreatectomy. In this study, DFAC was more reliable than DFAA as a predictor of CR‐POPF after pancreatectomy.

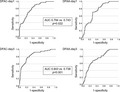

## 1 INTRODUCTION

Postoperative pancreatic fistula (POPF) remains one of the most common complications after pancreatic surgery, such as pancreaticoduodenectomy (PD) and distal pancreatectomy (DP). Despite modifications in surgical techniques and perioperative patient care to prevent POPF, the incidence of POPF has been reported to range from 3%–50%, even at high‐volume centers.^1^, ^2^, ^3^, ^4^

The International Study Group of Pancreatic Surgery (ISGPS) has developed and validated a universally applicable definition for POPF.^5^, ^6^ According to the classification, grade B or C POPF is regarded as clinically relevant POPF (CR‐POPF) and requires major deviations in clinical management. CR‐POPF can lead to more life‐threatening complications, including intraabdominal abscess or intraabdominal bleeding and septicemia. As a result, CR‐POPF is responsible for prolonged hospital stays and increased healthcare costs.

Many studies have demonstrated risk factors for CR‐POPF, such as pancreatic texture, pancreatic duct size, body mass index (BMI), and massive intraoperative blood loss.^7^, ^8^ Recently, several studies have reported that the drain fluid amylase concentration (DFAC) on the 1st and 3rd d after pancreatectomy can be a reliable predictor of CR‐POPF.^9^, ^10^, ^11^, ^12^, ^13^, ^14^, ^15^, ^16^, ^17^, ^18^ DFAC can reveal the optimal timing of drain removal after pancreatectomy by data‐driven decisions. However, the value of the drain fluid amylase amount (DFAA) when considering the daily drainage volume has not been investigated, so it is unclear whether DFAC or DFAA is the more reliable predictor of CR‐POPF after pancreatectomy. Therefore, this study aimed to investigate the practical significance of DFAA as a predictor of CR‐POPF following pancreatectomy.

## 2 PATIENTS AND METHODS

Between August 2014 and November 2020, 216 consecutive patients who underwent PD (n = 126) or DP (n = 90) at our institution were enrolled in the study. Patients undergoing hepatopancreaticoduodenectomy (n = 12), central pancreatectomy (n = 4), total pancreatectomy (n = 18), or partial pancreatectomy (n = 1) were excluded. The demographics, clinical characteristics, operative details, and postoperative outcomes of patients with and without CR‐POPF were retrospectively analyzed. All patients provided written informed consent before undergoing therapy. This study was approved by the Institutional Review Board of our institution (No. 2020‐119) and was performed in accordance with the 1964 Declaration of Helsinki and its later amendments or comparable ethical standards.

### 2.1 Surgical procedure

After review by a multidisciplinary board, all pancreatic disease cases were assessed by pancreatic surgeons to determine resectability and the most appropriate surgical procedure. The subtotal stomach‐preserving method was the standard procedure for PD. In patients with malignant disease, lymph nodes are dissected at the hepatoduodenal ligament, around the common hepatic artery, around the superior mesenteric artery (SMA), and around the pancreatic head. Transection of the pancreatic parenchyma was performed with an electric scalpel. A modified Child method, with duct‐to‐mucosa pancreatojejunostomy, was chosen for organ reconstruction in all cases. The modified Blumgart mattress suture was the procedure of choice for pancreatic remnant reconstruction when feasible.^19^, ^20^ In all cases, a 4‐Fr polyethylene tube was placed through the pancreatojejunal anastomotic site as an external stent. Three silastic flexible drains were routinely placed adjacent to the anastomosis and at both the cranial and caudal sites of the pancreatojejunostomy and choledochojejunostomy.

In the case of DP, transection of the pancreatic parenchyma was performed with a stapler from 2017 on. A silastic flexible drain was placed at the pancreatic stump. However, in the case of radical antegrade modular pancreatosplenectomy (RAMPS) for pancreatic ductal adenocarcinoma (PDAC), an additional drain was placed at the left subphrenic space.

Energy devices, such as LigaSure (TM) (Covidien, Japan) was used only during laparoscopic surgery. All drains were closed but not suctioned. Prophylactic octreotide to prevent POPF was not administered in either PD or DP.

### 2.2 Definition of DFAC and DFAA

Amylase levels in the drainage fluid were routinely measured on postoperative d (PODs) 1 and 3 (ie, DFAC‐day 1 and DFAC‐day 3). If there were multiple drains, the highest DFAC‐day 1 value was defined as analysis drain. DFAA‐day 1 was calculated by multiplying DFAC‐day 1 and the 24‐h drainage volume from the morning of POD 1. In the same way, DFAA‐day 3 was calculated by multiplying DFAC‐day 3 and the 24‐h drainage volume from the morning of POD 3.

The drain was removed on POD 4 or 5 if the drainage fluid was clear and pancreatic fistula and bacterial contamination were absent. In the case that bacterial contamination was detected based on a bacterial culture test of drain fluid, or suspected by nonserous (turbid) fluid of the drain, drains were replaced on POD 7.

### 2.3 Postoperative complications

Any complications that developed within 90 d after the operation were included. No patients were lost to follow‐up. CR‐POPF included grade B or C POPF based on the definition of ISGPS.^6^ Bile leakage was defined according to International Study Group of Liver Surgery (ISGLS) criteria.^21^ Intraabdominal bleeding and delayed gastric emptying (DGE) were also defined by ISGPS criteria.^22^, ^23^ Surgical mortality was defined as perioperative death within the first 90 d following surgery.

### 2.4 Statistical analysis

The continuous data are expressed as the medians (ranges). The statistical analyses were performed using chi square tests, Mann–Whitney *U*‐tests, or Fisher's exact probability tests, as appropriate. The predictive ability of DFAC and DFAA for the occurrence of CR‐POPF was assessed by calculating the area under the receiver operating characteristic (ROC) curve. The variables identified as potentially significant by univariate analysis were selected for multivariate analysis with a logistic regression model to identify the independent predictors of CR‐POPF. All *P* values were 2‐sided, and *P *< .05 was considered to indicate a statistically significant difference. All statistical calculations were performed using the IBM SPSS Statistics 27 software package (IBM Japan).

## 3 RESULTS

### 3.1 Patient characteristics and surgical outcomes

The patient demographics and clinical characteristics are shown in Table 1. CR‐POPF was found in 75 patients (35%). Among the CR‐POPF patients, grade C POPF occurred in only two patients (3%) who underwent PD, and the remaining 73 patients (97%) had grade B POPF. The CR‐POPF group had a significantly higher BMI than the no CR‐POPF group (*P* < .001). The CR‐POPF group had a significantly lower incidence of PDAC than the no CR‐POPF group (*P* < .001). The median age, sex, preoperative albumin concentration, and incidence of comorbidities did not differ between the two groups.

**TABLE 1:** Patient characteristics

	CR‐POPF		*P*
	No (n = 141)	Yes (n = 75)	
Age (y)	70 (28–89)	69 (24–88)	.147
Sex (male/female)	62/79	40/35	.201
BMI (kg/m^2^)	20.8 (13.3–35.0)	22.8 (14.6–32.0)	<.001
Albumin (g/dL)	3.6 (2.4–4.8)	3.8 (1.7–4.8)	.103
ASA status (I/II/III)	83/57/1	56/17/2	.021
Comorbidity			
Cardiovascular disease	17 (12%)	7 (9%)	.652
Cerebrovascular disease	8 (6%)	1 (1%)	.167
Pulmonary disease	10 (7%)	6 (8%)	.791
Chronic kidney disease	7 (5%)	3 (4%)	1.000
Diabetes mellitus	36 (26%)	11 (15%)	.083
Previous intraabdominal operation	23 (16%)	8 (11%)	.312
Disease			
PDAC	81 (57%)	22 (29%)	<.001
Cancer excepting PDAC^a^	15 (11%)	17 (23%)	
IPMN	15 (11%)	6 (8%)	
NET	6 (4%)	10 (13%)	
Others	24 (17%)	20 (27%)	

Note

Expressed as N (%) or median (range).

Abbreviations: ASA, American Society of Anesthesiologists; BMI, body mass index; CR‐POPF, clinically relevant postoperative pancreatic fistula; IPMN, intraductal papillary mucinous neoplasm; NET, neuroendocrine tumor, PDAC, pancreatic ductal adenocarcinoma.

^a^ Includes duodenal, ampullary of the pancreas, and bile duct cancer.

The laparoscopic approach was performed in 24 cases during DP. Among the 126 cases of PD, the incidence of patients who underwent the modified Blumgart method was 79% (100 cases). Table 2 shows the surgical outcomes after pancreatectomy. The type of pancreatectomy, operative duration, and total blood loss volume did not differ between the two groups. There were significant differences between the two groups in terms of the pancreatic duct size <3 mm (53% in the no CR‐POPF group vs 85% in the CR‐POPF group) and soft pancreatic texture rate (62% in the no CR‐POPF group vs 80% in the CR‐POPF group). The median DFAC‐day 1, DFAA‐day 1, DFAC‐day 3, and DFAA‐day 3 were significantly higher in the CR‐POPF group than in the no CR‐POPF group. On the other hand, the median drainage volumes on POD 1 and POD 3 were significantly lower in the CR‐POPF group than in the no CR‐POPF group (Figure [Link], [Link]). There were significant differences between the two groups in terms of intraabdominal bleeding (0% in the no CR‐POPF group vs 5% in the CR‐POPF group) and median postoperative hospital stays (12 d in the no CR‐POPF group vs 30 d in the CR‐POPF group). The in‐hospital and 90‐d postoperative mortality rates were zero in both groups.

**TABLE 2:** Surgical outcome

	CR‐POPF		*P*
	No (n = 141)	Yes (n = 75)	
Type of pancreatectomy			
Pancreaticoduodenectomy	88 (70%)	38 (30%)	.111
Distal pancreatectomy	53 (59%)	37 (41%)	
Operative duration (min)	334 (124–660)	362 (109–598)	.944
Total blood loss (mL)	320 (3–4514)	458 (3–4250)	.324
Pancreatic duct size (<3 mm)	75 (53%)	64 (85%)	<.001
Pancreatic texture			
Soft	87 (62%)	60 (80%)	.006
Hard	54 (38%)	15 (20%)	
Postoperative day 1 drain status			
DFAC (U/L)	981 (11–32 048)	5559 (90–30 017)	<.001
Drainage volume (mL)	54 (3–658)	39 (3–740)	.018
DFAA (U)	50 (1–2832)	180 (8–2014)	<.001
Postoperative day 3 drain status			
DFAC (U/L)	268 (8–14 500)	3271 (31–142 458)	<.001
Drainage volume (mL)	42 (1–633)	15 (1–352)	<.001
DFAA (U)	11 (1–499)	36 (3–712)	<.001
Morbidity other than CR‐POPF			
Bile leakage	3 (2%)	1 (1%)	1.000
Intraabdominal abscess	14 (10%)	3 (4%)	.184
Intraabdominal bleeding	0	5 (7%)	.005
Delayed gastric emptying	8 (6%)	2 (3%)	.724
Septicemia	1 (1%)	3 (4%)	.122
Mortality	0	0	—
Postoperative hospital stays (d)	12 (6–138)	30 (13–90)	<.001

Note

Expressed as N (%) or median (range).

Abbreviations: CR‐POPF, clinically relevant postoperative pancreatic fistula; DFAA, drain fluid amylase amount; DFAC, drain fluid amylase concentration.

### 3.2 Cutoff values of DFAC/DFAA‐day 1 and day 3 for predicting CR‐POPF

**FIGURE 1:**
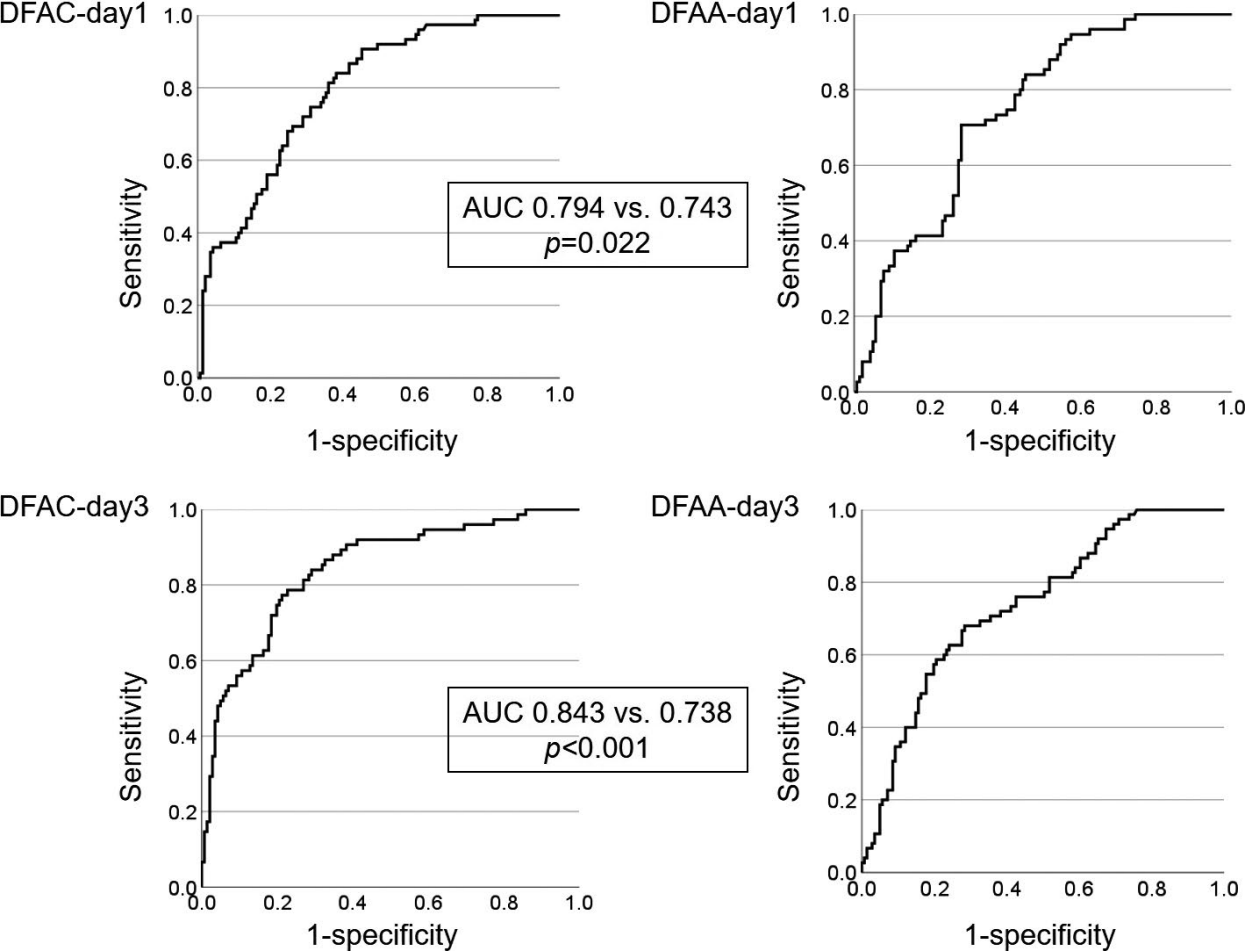
ROC curve of DFAC/DEAA‐day 1 and day 3 for rates of CR‐POPF for all patient groups

The ROC curves for generating cutoff values of DFAC/DEAA‐day 1 and day 3 for rates of CR‐POPF for all patient groups are shown in Figure 1 and Table 3. The cutoff value of DFAC‐day 1 was 1757 U/L, with a sensitivity of 84%, specificity of 62%, and accuracy of 69%. The cutoff value of DFAA‐day 1 was 139 U, with a sensitivity of 71%, specificity of 72%, and accuracy of 71%. The cutoff value of DFAC‐day 3 was 1044 U/L, with a sensitivity of 73%, specificity of 79%, and accuracy of 78%. The cutoff value of DFAA‐day 3 was 21 U, with a sensitivity of 68%, specificity of 72%, and accuracy of 70%. Altogether, the results indicated that the most reliable predictor of CR‐POPF after pancreatectomy was DFAC‐day 3, which had the highest area under the ROC curve (AUC) value (0.843; optimal cutoff value 1044 U/L).

**TABLE 3:** Accuracy of DFAC/DFAA‐day 1 and day 3 to predict CR‐POPF

Variable	Cutoff value	AUC	Sensitivity	Specificity	PPV	NPV	Accuracy
DFAC‐day 1	1757 (U/L)	0.794	84%	62%	54%	88%	69%
DFAA‐day 1	139 (U)	0.743	71%	72%	57%	82%	71%
DFAC‐day 3	1044 (U/L)	0.843	73%	79%	66%	87%	78%
DFAA‐day 3	21 (U)	0.738	68%	72%	56%	81%	70%

Abbreviations: AUC, area under the ROC curve; CR‐POPF, clinically relevant postoperative pancreatic fistula; DFAA, drain fluid amylase amount; DFAC, drain fluid amylase concentration; NPV, negative predictive value; PPV, positive predictive value.

### 3.3 Subgroup analysis of DFAC/DFAA according to the type of pancreatectomy and predictors of CR‐POPF

Subgroup analysis of CR‐POPF according to the type of pancreatectomy was performed. The median operative duration was 424 min, and total blood loss was 495 mL following PD. The median operative duration was 245 min, and total blood loss was 178 mL following DP. CR‐POPF was found in 38 patients (30%) after PD and 37 patients (41%) after DP (*P* = .111). The ROC curves for generating cutoff values of DFAC/DEAA‐day 1 and day 3 for rates of CR‐POPF after PD are shown in Figure S2 and Table 4. The most reliable predictors of CR‐POPF after PD were DFAC‐day 1 (AUC = 0.855; optimal cutoff value 1773 U/L) and DFAC‐day 3 (AUC = 0.855; optimal cutoff value 713 U/L). The multivariate analysis results indicated that pancreatic duct size <3 mm (odds ratio [OR]: 4.20, 95% confidence interval [CI]: 1.47–11.94, *P* = .007) and DFAC‐day 3 value >713 U/L (OR: 15.66, 95% CI: 5.38–45.60, *P* < .001) were independently associated with CR‐POPF after PD (Table 5).

**TABLE 4:** Accuracy of DFAC/DFAA‐day 1 and day 3 to predict CR‐POPF after PD

Variable	Cutoff value	AUC	Sensitivity	Specificity	PPV	NPV	Accuracy
DFAC‐day 1	1773 (U/L)	0.855	84%	80%	64%	92%	81%
DFAA‐day 1	67 (U)	0.793	79%	71%	54%	89%	73%
DFAC‐day 3	713 (U/L)	0.855	84%	80%	64%	92%	81%
DFAA‐day 3	13 (U)	0.754	74%	69%	51%	86%	71%

Abbreviations: AUC, area under the ROC curve; CR‐POPF, clinically relevant postoperative pancreatic fistula; DFAA, drain fluid amylase amount; DFAC, drain fluid amylase concentration; NPV, negative predictive value; PD, pancreaticoduodenectomy; PPV, positive predictive value.

**TABLE 5:** Uni‐ and multivariate predictors of CR‐POPF after PD

Variable	Univariate			Multivariate		
	OR	95% CI	*P*	OR	95% CI	*P*
Age (y)						
>70 (n = 73)	1.66	0.56–4.90	.357			
<70 (n = 53)	1					
Sex						
Female (n = 70)	1.21	0.40–3.63	.740			
Male (n = 56)	1					
BMI (kg/m^2^)						
>24 (n = 36)	2.38	0.76–7.45	.138			
<24 (n = 90)	1					
Operative duration (min)						
>480 (n = 35)	1.89	0.46–7.74	.378			
<480 (n = 91)	1					
Total blood loss (mL)						
>1000 (n = 24)	1.97	0.38–10.28	.422			
<1000 (n = 102)	1					
Pancreatic duct size (mm)						
<3 (n = 58)	3.74	1.07–13.12	.039	4.20	1.47–11.94	.007
>3 (n = 68)	1			1		
Pancreatic texture						
Soft (n = 73)	1.49	0.39–5.67	.557			
Hard (n = 53)	1					
DFAA‐day 3 (U)						
>13 (n = 54)	0.734	0.21–2.57	.628			
<13 (n = 72)	1					
DFAC‐day 3 (U/L)						
>713 (n = 50)	27.36	6.00–124.74	<.001	15.66	5.38–45.60	<.001
<713 (n = 76)	1			1		

Abbreviations: BMI, body mass index; CR‐POPF, clinically relevant postoperative pancreatic fistula; DFAA, drain fluid amylase amount; DFAC, drain fluid amylase concentration; PD, pancreaticoduodenectomy.

The ROC curves for generating cutoff values of DFAC/DEAA‐day 1 and day 3 for rates of CR‐POPF after DP are shown in Figure S3 and Table 6. The most reliable predictor of CR‐POPF after DP was DFAC‐day 3 (AUC = 0.819; optimal cutoff value 3506 U/L). The multivariate analysis results indicated that an operative duration >300 min (OR: 3.79, 95% CI: 1.04–13.86, *P* = .044), total blood loss >400 mL (OR: 3.63, 95% CI: 1.03–12.72, *P* = .044), and DFAC‐day 3 value ≥3506 U/L (OR: 40.57, 95% CI: 9.80–167.92, *P* < .001) were independently associated with CR‐POPF after DP (Table 7).

**TABLE 6:** Accuracy of DFAC/DFAA‐day 1 and day 3 to predict CR‐POPF after DP

Variable	Cutoff value	AUC	Sensitivity	Specificity	PPV	NPV	Accuracy
DFAC‐day 1	9401 (U/L)	0.715	51%	94%	86%	74%	77%
DFAA‐day 1	139 (U)	0.674	78%	55%	55%	78%	64%
DFAC‐day 3	3506 (U/L)	0.819	62%	93%	85%	78%	80%
DFAA‐day 3	21 (U)	0.711	76%	66%	61%	80%	70%

Abbreviations: AUC, area under the ROC curve; CR‐POPF, clinically relevant postoperative pancreatic fistula; DFAA, drain fluid amylase amount; DFAC, drain fluid amylase concentration; DP, distal pancreatectomy; NPV, negative predictive value; PPV, positive predictive value.

**TABLE 7:** Uni‐ and multivariate predictors of CR‐POPF after DP

Variable	Univariate			Multivariate		
	OR	95% CI	*P*	OR	95% CI	*P*
Age (y)						
>70 (n = 39)	0.67	0.19–2.38	.553			
<70 (n = 51)	1					
Sex						
Female (n = 44)	0.82	0.18–3.74	.799			
Male (n = 46)	1					
BMI (kg/m^2^)						
>24 (n = 19)	0.85	0.20–3.67	.827			
<24 (n = 71)	1					
Stump closure method						
Stapler (n = 49)	0.43	0.11–1.64	.217			
Handsewn (n = 41)	1					
Operative duration (min)						
>300 (n = 20)	3.90	0.95–15.97	.059	3.79	1.04–13.86	.044
<300 (n = 70)	1			1		
Total blood loss (mL)						
>400 (n = 25)	2.33	0.49–11.09	.290	3.63	1.03–12.72	.044
<400 (n = 65)	1			1		
Pancreatic duct size (mm)						
<3 (n = 9)	1.67	0.20–13.72	.634			
>3 (n = 81)	1					
Pancreatic texture						
Soft (n = 74)	0.63	0.14–2.73	.533			
Hard (n = 16)	1					
DFAA‐day 3 (U)						
>21 (n = 46)	2.12	0.51–8.72	.299			
<21 (n = 44)	1					
DFAC‐day 3 (U/L)						
>3506 (n = 27)	23.31	4.61–117.85	<.001	40.57	9.80–167.92	<.001
<3506 (n = 63)	1			1		

Abbreviations: BMI, body mass index; CR‐POPF, clinically relevant postoperative pancreatic fistula; DFAA, drain fluid amylase amount; DFAC, drain fluid amylase concentration; DP, distal pancreatectomy.

## 4 DISCUSSION

Pancreatic surgery has become safer, especially in high‐volume centers, due to advances in technology and perioperative management. Nevertheless, CR‐POPF is the most common complication and subsequently triggers other infectious complications, which is concerning for both patients and surgeons. Many studies have reported risk factors for CR‐POPF, including not only pancreas‐related factors (eg, pancreatic texture and pancreatic duct size) but also perioperative factors (eg, massive intraoperative blood loss and DFAC).^7^, ^8^, ^9^, ^10^, ^11^, ^12^, ^13^, ^14^, ^15^, ^16^, ^17^, ^18^

DFAC can help to determine the optimal timing of drain removal after pancreatectomy with data‐driven decisions. However, various DFAC cutoff values have been proposed in the literature. Molinari et al^15^ demonstrated that a DFAC‐day 1 value ≥5000 U/L was a significant predictive factor of POPF after PD and DP. Ansorge et al^17^ reported that a DFAC‐day 1 value ≥1322 U/L was a significant predictive factor of CR‐POPF after PD. Maggino et al^10^ reported that a DFAC‐day 1 value ≥2000 U/L was a significant predictive factor of CR‐POPF after DP. According to the systematic review and meta‐analysis by Yang et al,^18^ a DFAC‐day 1 value ≥1300 U/L was a risk factor for POPF after pancreatectomy. Few studies have reported DFAC‐day 3 data in predicting CR‐POPF^12^, ^13^. Noji et al^12^ showed that a DFAC‐day 3 value ≥3000 U/L was the best cutoff value of CR‐POPF after pancreaticoenteral anastomosis, and DFAC‐day 3 was more useful than DFAC‐day 1. In our study, the most reliable predictor of CR‐POPF after pancreatectomy was DFAC‐day 3, which had the highest area under the ROC curve (AUC) value (0.843; optimal cutoff value 1044 U/L). Thus, the best cutoff value of the DFAC remains unclear, but once identified, it can provide a reference for institution‐specific early drain removal strategies.

Even with the same surgery, postoperative drainage output varies from case to case. If the drainage volume is large, including serous ascites, DFAC will be diluted and therefore lowered. Prior to this study, we considered DFAC to be of limited utility because the drainage quantity was not being considered. However, the results of this study revealed DFAC to be more reliable than DFAA as a predictor of CR‐POPF after pancreatectomy. One of the reasons for this result was the difference in the drainage volume between the CR‐POPF group and the no CR‐POPF group. The median drainage volumes on POD 1 and POD 3 were significantly lower in the CR‐POPF group than in the no CR‐POPF group. The exact reason for this is unclear, but the CR‐POPF group was more turbid, and the drainage efficiency by nonsuctioned (gravity) drains may have been low. Leakage of the pancreatic juice potentially elicits local inflammatory changes around the pancreatic stump or pancreatojejunostomy, which is related to the dense adhesion of the surrounding tissue. This may be the cause of the difference in fluid volume between the cases with and those without CR‐POPF. On the other hand, Molinari et al^15^ reported that there was no significant difference in drainage volume between the POPF group and the no POPF group following pancreatectomy. Further investigation into the drainage volume after pancreatectomy based on the type and method of drainage is needed.

In this study, subgroup analysis of CR‐POPF according to the type of pancreatectomy was performed. The cutoff value of DFAC‐day 3 after PD was 713 U/L, while the cutoff value of DFAC‐day 3 after DP was 3506 U/L. Previous reports have also tended to report higher cutoff values for DP than for PD.^10^, ^12^, ^15^, ^17^ Since DP does not include pancreaticoenteral anastomosis, CR‐POPF may not develop, even if a biochemical leak occurs. Therefore, the drain removal strategy should be adjusted according to the surgical procedure. The patients with normal DFAC‐day1 level after DP may be able to remove the drain within POD 1, because of DP is less likely to have more serious complications than PD.

Although early drain removal following pancreatectomy resulted in a reduction in CR‐POPF, most surgeons continued to remove drains late, even when DFAC was low, which was associated with inferior outcomes. Seykora et al^24^ reported that the early drain removal (on or before POD 3) rate after DP was only 15.2% in a retrospective cohort study of the American College of Surgeons' National Surgical Quality Improvement Program (ACS‐NSQIP) registry. Additionally, DFAC was not assessed in most cases by surgeons who removed drains late. Some surgeons believe they will control POPF and intraabdominal abscess via drain placement following pancreatic resection. However, prolonged drains interfere with adjacent structures and introduce bacteria into sterile pancreatic fluid collection, increasing the incidence of CR‐POPF and intraabdominal abscesses. Although both pancreas‐related risk factors and perioperative risk factors, including DFAC, should be considered, serous (nonturbid) fluid visible to the naked eye indicates that the drain should be removed as soon as possible. On the other hand, Ohgi et al^25^ reported that latent pancreatic fistula (LPF) with initially normal DFAC occurred 10%, and LPF was significantly associated with severe complication and worse outcome after PD.

This study has some limitations that should be mentioned. First, this was a single‐center retrospective study, and a propensity score matching analysis was not possible because the number of patients with CR‐POPF was too small. However, the strength of this study is that surgical techniques and postoperative management were unified. Second, the cutoff values of DFAC and DFAA were derived from nonsuctioned drains. There were no data on the type of drain suction used after pancreatectomy in our institution. However, Kone et al^26^ recently analyzed closed suction vs closed gravity drainage after pancreatectomy in a large sample. They showed that the type of drain is not associated with increased CR‐POPF or other postoperative outcomes. In the future, large‐series multicenter studies evaluating the clinical impact of DFAC will help to compensate for the limitations of this study.

In conclusion, the results of our study indicate that DFAC is more reliable than DFAA as a predictor of CR‐POPF after pancreatectomy. Routine postoperative assessment of DFAC could provide more meticulous follow‐up after pancreatectomy. DFAC can indicate the optimal timing of drain removal after pancreatectomy. However, the cutoff value of DFAC may vary slightly between institutions. Therefore, we propose an early drain removal strategy based on institution‐specific DFAC values, along with consideration of other risk factors for CR‐POPF.

## DISCLOSURE

Funding: The author(s) received no financial support for the research, authorship, and/or publication of this article.

Ethics Approval and Consent to Participate: This study was approved by the Institutional Review Board of Aichi Medical University (No. 2020–119) and performed in accordance with the 1964 Declaration of Helsinki and its later amendments or comparable ethical standards. All patients provided signed informed consent before surgery.

Conflict of Interest: The authors have no conflicts of interest to disclose.

Author Contributions: Fukami designed the study and wrote the initial draft of the article. Sano contributed to interpretation of the data and the critical revision of the article for important intellectual content. All the other authors (TS, TO, TH, TK, SK, TM, SK, and KK) contributed to the data collection and interpretation and critically reviewed the article. All the authors have read and approved the final version of the article and have agreed to be accountable for all aspects of the study, ensuring that any questions related to the accuracy or integrity of any part of the work are resolved.

## Supporting information

Fig S1a

Fig S1b

Fig S2

Fig S3
