# Supplementary figures and images for: Which is the best predictor of clinically relevant pancreatic fistula after pancreatectomy: drain fluid concentration or total amount of amylase?
Source: Ann Gastroenterol Surg. 2021 May 11;5(6):844–52. doi: 10.1002/ags3.12471 (PMC8560612; doi:10.1002/ags3.12471)

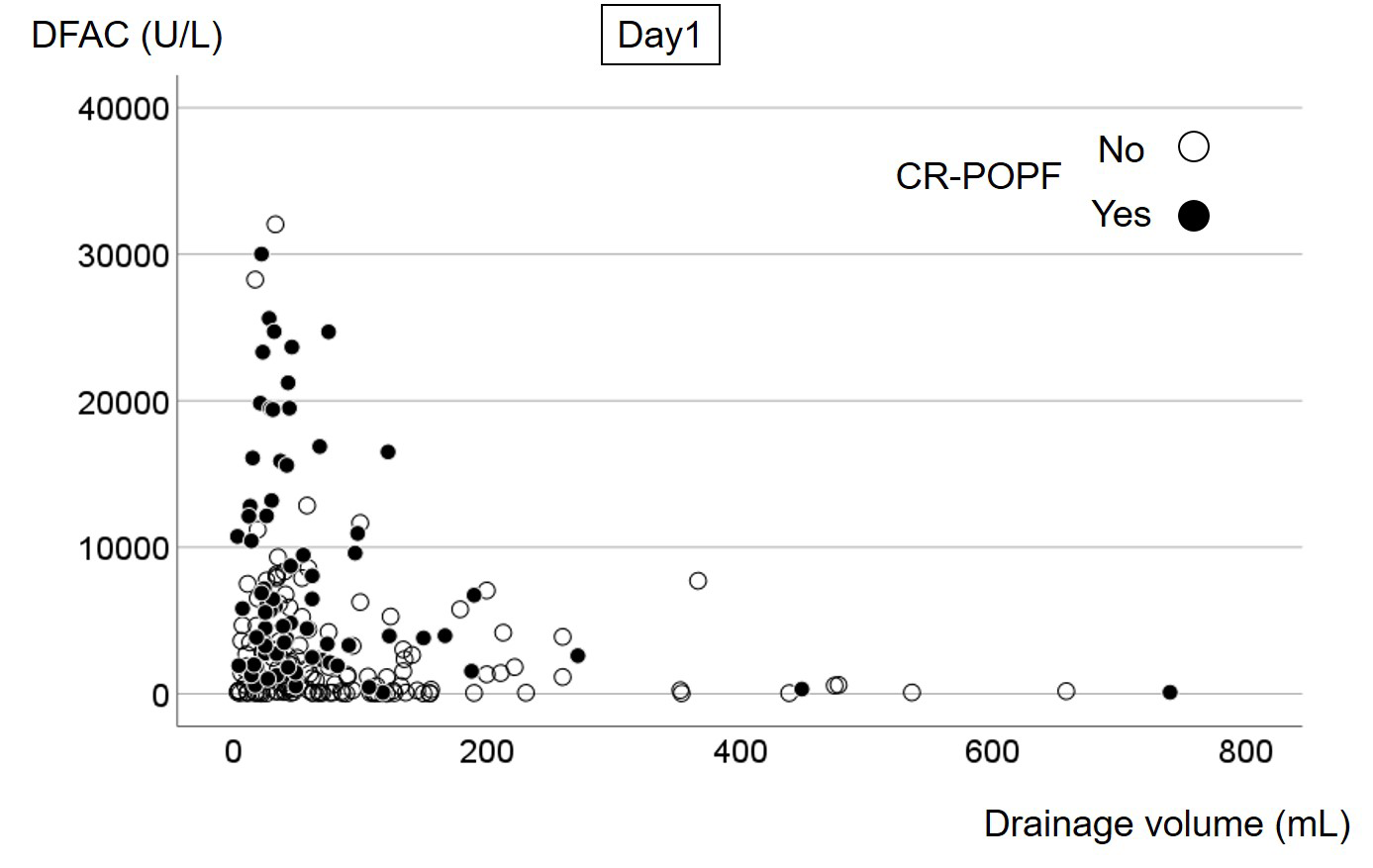

Supplement: Supplementary file 1 — Fig S1a [file AGS3-5-844-s004.tif]

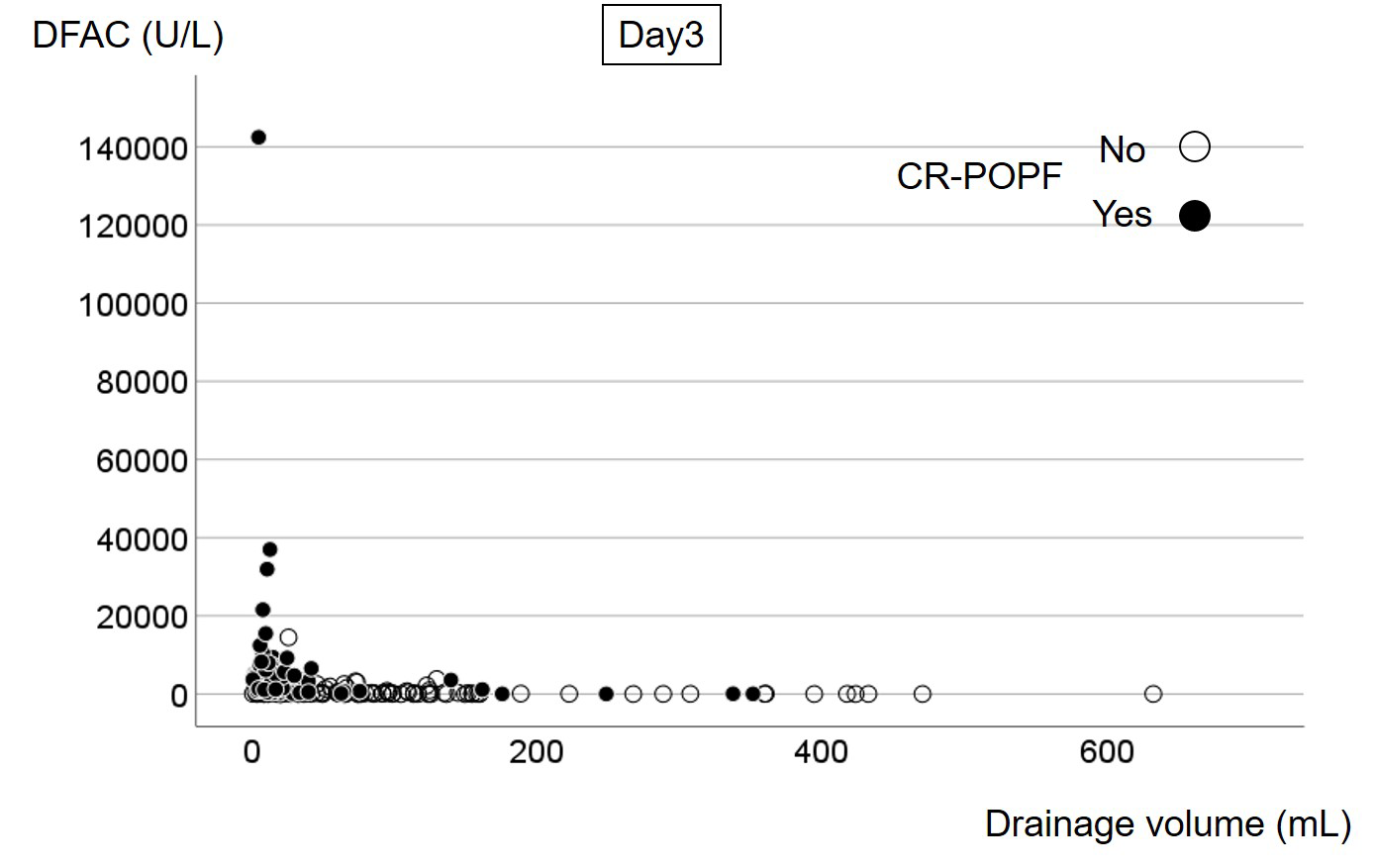

Supplement: Supplementary file 2 — Fig S1b [file AGS3-5-844-s003.tif]

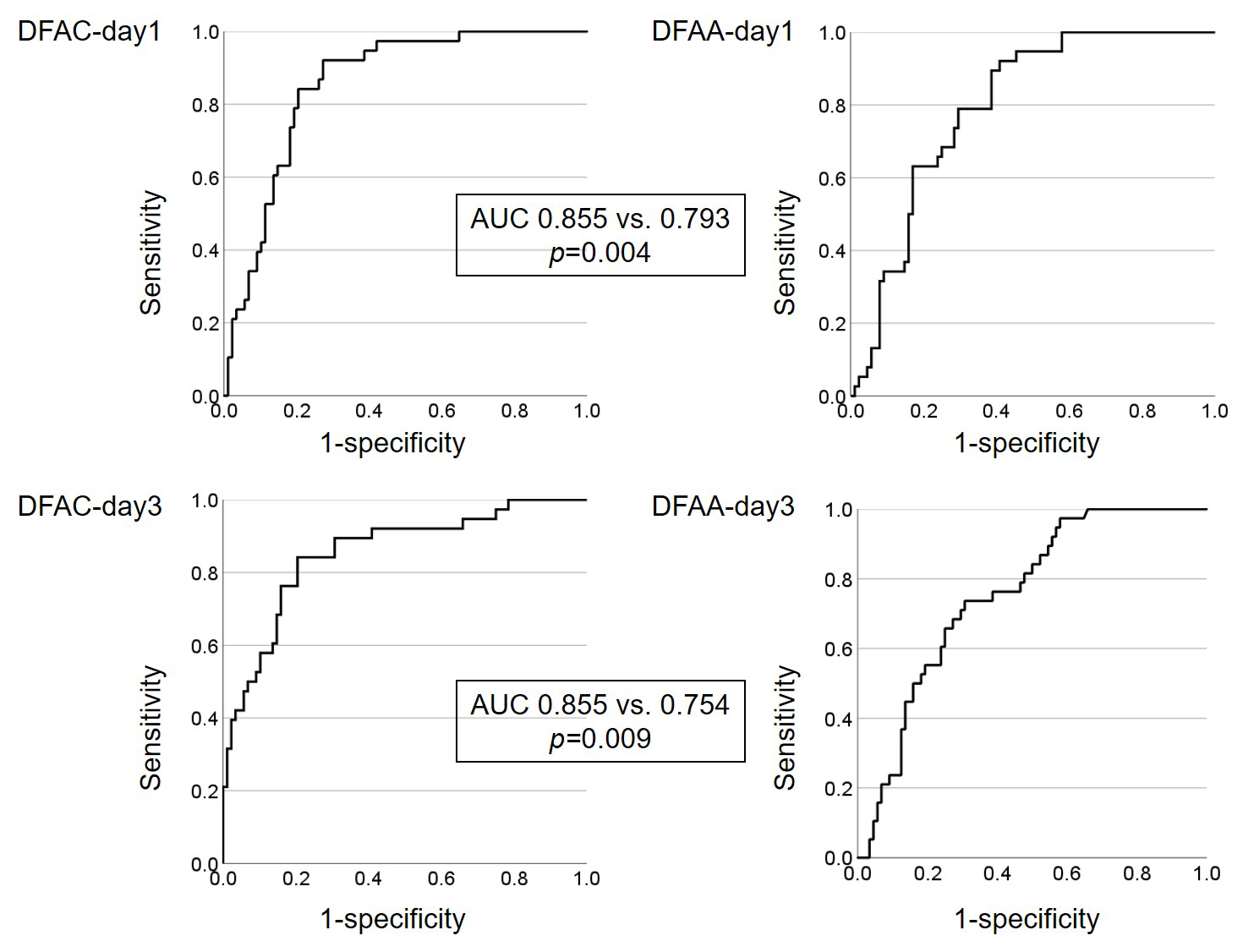

Supplement: Supplementary file 3 — Fig S2 [file AGS3-5-844-s001.tif]

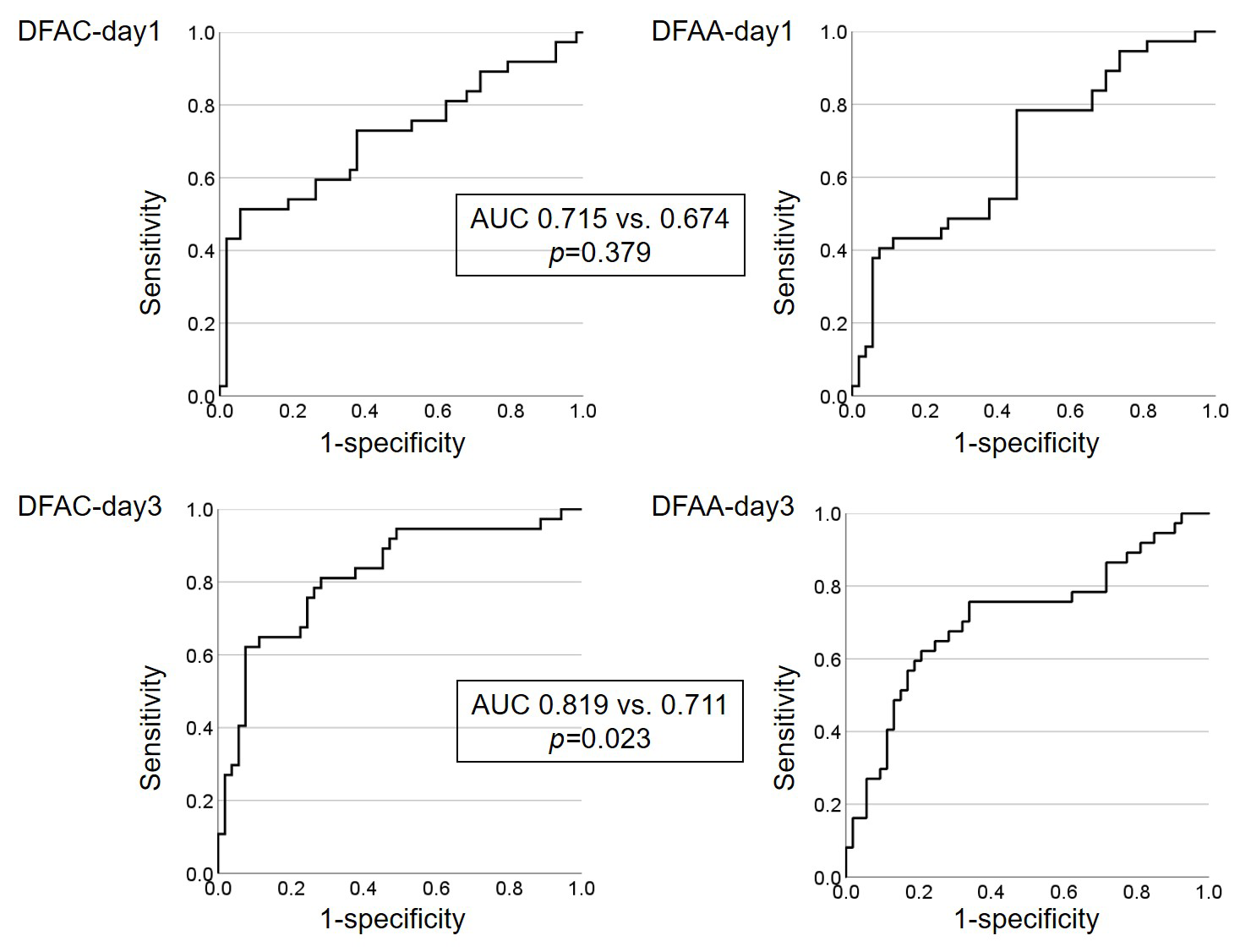

Supplement: Supplementary file 4 — Fig S3 [file AGS3-5-844-s002.tif]
